# Supplementary material for: Indications of food insecurity in the content of telephone calls to a community referral system
Source: PLoS One. 2023 Apr 20;18(4):e0284624. doi: 10.1371/journal.pone.0284624 (PMC10118074; doi:10.1371/journal.pone.0284624)
Supplement: Attachment — Submitted filename: Response to Reviewers.docx [file pone.0284624.s001.docx]

Thank you for the opportunity to revise and resubmit our manuscript (PONE-D-22-22201), entitled “Indications of food insecurity in the content of telephone calls to a community referral system” for reconsideration by *PLOS ONE*.

We thank the editor and reviewers for their useful comments. We appreciate the opportunity to address the comments by submitting a revision. We have considered all comments and believe that the revisions have addressed these comments and greatly improved the paper. We have also changed the title to comply with Reviewer 1’s suggestion.

Below, we have addressed each reviewer’s concerns by explaining in detail the responses (**in** **bold**) to their questions/concerns (numbered), and highlighting the changes in the manuscript with Track Changes. We also refer the reviewers to different line numbers within the “Revised Manuscript with Trach Changes” document. In addition, we carefully responded to the *PLOS ONE* Revision Guide.

# Journal Requirements

1. Please ensure that your manuscript meets PLOS ONE's style requirements, including those for file naming. The PLOS ONE style templates can be found at

<https://journals.plos.org/plosone/s/file?id=wjVg/PLOSOne_formatting_sample_main_body.pdf> and

<https://journals.plos.org/plosone/s/file?id=ba62/PLOSOne_formatting_sample_title_authors_affiliations.pdf>

**We complied with this request.**

2. Please ensure that you have specified (1) whether consent was informed and (2) what type you obtained (for instance, written or verbal, and if verbal, how it was documented and witnessed). If your study included minors, state whether you obtained consent from parents or guardians. If the need for consent was waived by the ethics committee, please include this information.

**Please see the Methods section, Line 88.**

3. We note that you have indicated that data from this study are available upon request. PLOS only allows data to be available upon request if there are legal or ethical restrictions on sharing data publicly. For more information on unacceptable data access restrictions, please see <http://journals.plos.org/plosone/s/data-availability#loc-unacceptable-data-access-restrictions>.

**The full list of quotes is available by request from the authors only if the requester signs a Data Sharing Agreement with the United Way of Salt Lake. This data is owned by a third-party organization and we do not have the right to share them. We have provided their contact information in the cover letter.**

4. In your revised cover letter, please address the following prompts:
   a. If there are ethical or legal restrictions on sharing a de-identified data set, please explain them in detail (e.g., data contain potentially sensitive information, data are owned by a third-party organization, etc.) and who has imposed them (e.g., an ethics committee). Please also provide contact information for a data access committee, ethics committee, or other institutional body to which data requests may be sent.
   b. If there are no restrictions, please upload the minimal anonymized data set necessary to replicate your study findings as either Supporting Information files or to a stable, public repository and provide us with the relevant URLs, DOIs, or accession numbers. For a list of acceptable repositories, please see <http://journals.plos.org/plosone/s/data-availability#loc-recommended-repositories>.

**Please see the updated cover letter.**

# Reviewer #1 (Elisabeth Garratt)

1. While interesting enough, I am not recommending this paper to be accepted for publication in PLOS One, for two key reasons.
   a. First, by reporting on a small study with a specific geographical context, the scope of its contribution is small, and insufficient to merit inclusion in an international journal such as PLOS One. When reworking this paper for submission elsewhere I would encourage the authors to think carefully about how their findings might have relevance beyond the immediate research context and bring this out more clearly in the revised work.
   b. Second, I have reservations about the paper’s scholarly rigor, especially the analyses, which identified overlapping analytical themes and thereby felt weak and lacking robustness.

**We acknowledge your concern and have provided more content (e.g., details about themes, the generalizability of findings, sample selection, the role of 211) to the manuscript to comply with your feedback.**

**Our analysis provides a clear answer to a simple and important question: Are calls to a community helpline for food assistance a good proxy for food insecurity? The qualitative analysis reported in this paper uses unique data coming from a partnership between the first author and a community organization—United Way of Salt Lake, which operates the 211 system in Utah. The analysis extracted information that was hidden in the contents of food-related information seeking calls and highlighted indications of food insecurity through these calls.**

**Our sample size is similar to other qualitative analyses published in PLOS ONE and other high-impact journals (please see the below publications) and it is within a reasonable range for qualitative analysis. For instance, Derose et al [1] interviewed 30 women living with HIV to examine factors contributing to food insecurity; Speirs et al [2] interviewed 15 individuals in a caring role for someone with bipolar disease to learn about their experiences, or Agarwal et al [3] interviewed 26 primary care practitioners to identify factors contributing to burnout.**

[1] [**https://journals.plos.org/plosone/article?id=10.1371/journal.pone.0181568**](https://journals.plos.org/plosone/article?id=10.1371/journal.pone.0181568)

[2] [**https://journals.plos.org/plosone/article?id=10.1371/journal.pone.0280059**](https://journals.plos.org/plosone/article?id=10.1371/journal.pone.0280059)

[3] [**https://jamanetwork.com/journals/jamainternalmedicine/fullarticle/2758330**](https://jamanetwork.com/journals/jamainternalmedicine/fullarticle/2758330)

[4] [**https://bjgpopen.org/content/4/5/bjgpopen20X101143**](https://bjgpopen.org/content/4/5/bjgpopen20X101143)

**In addition, since the 211 network is available in U.S. and Canada and is widely used, this finding has practical implications for the surveillance of food insecurity. Specifically, 211 is available to approximately 309 million people in the U.S. and connects people to local services.**

**To address your second concern, we provided more information in the Methods section. We also addressed your concerns numbered 16 and 17.**

2. The title ‘Real-time indications of food insecurity in telephone calls to a community referral system’ is not an accurate representation of the focus of this paper, as the paper contains no temporal element (such as how calls to the community referral system have changed over a given time period, or fluctuations over the course of a month). I would like to see the title provide a closer reflection of the paper’s focus and scope.

**Thank you for bringing this issue to our attention. We changed the title to “Indications of food insecurity in the content of telephone calls to a community referral system”.**

3. Linking natural disasters to food security (p2) is an important consideration in the Global South but its relevance to the Global North (where this study is situated) is not clear.

**The 211 network is available in the US and Canada, where many natural disasters happen every year including wildfires, hurricanes, and the ongoing COVID-19 pandemic. All these natural disasters have been linked to disruptions in the food supply. Please see line 52.**

4. The sentence ‘Lack of donations to food pantries [7] and the increasing rate of inflation [8] make it harder for food pantries to meet the demand and drive them to closure, which will put people who rely on emergency food resources at risk of food insecurity’ (p2) misunderstands the nature of food insecurity and the role of food banks in assisting those experiencing food insecurity. People who rely on emergency food are almost by definition experiencing food insecurity, and the inadequacy of food banks in alleviating food insecurity is well documented. It is therefore inaccurate to talk of food banks as protecting people from food insecurity.

**Although food pantry users are more likely to experience food insecurity compared to non-pantry users, not all pantry users are food insecure. In addition, findings on the role of food pantries in alleviating food insecurity are mixed. According to the below publication, “food banks have the potential to improve food security outcomes when operational resources are adequate, provisions of perishable food groups are available, and client needs are identified and addressed.” However, when the needs of people, either food-secure or food-insecure, who rely on food pantries cannot be met, they have to find another source of emergency food.**

<https://link.springer.com/article/10.1007/s10900-015-0147-5>

5. More contextual information is needed about the 211 network (p3). In particular, what is its purpose? Who is it funded by? Does it offer solely information or is it a means of callers being referred to relevant services? And who are United Way? Your international readers may not have heard of this organisation or be familiar with its goals and purpose of operation.

**Thank you for calling attention to this problem.**

**For the purpose of 211, please see lines 63-64, which reads as “The 211 network has the most comprehensive source of information about local community resources, helping people across the U.S. and Canada by providing information and referrals to social assistance programs.”**

**211 is funded by the states and donations. See line 68.**

**Callers receive the information they need directly from 211 and sometimes callers can share their contact information, so that community organizations can reach out to them.**

**United Way is a privately funded charity, actively working in 95% of U.S. communities to improve education, economy, and health. Please see lines 89-89.**

6. The authors state that ‘Researchers have also used food-related calls to 211 as a proxy for food insecurity’. What is the value for doing so? What can it add to national prevalence estimates, for example? Unpacking this point would help the authors demonstrate the value of this study more convincingly.

**211 calls are reported at the ZIP Code level, allowing researchers to conduct granular analysis. This is very valuable as food insecurity estimates are at the national and state level. In addition, 211 reports data daily while the national estimates are reported annually. Another organization, Feeding America, has used these estimates to measure county-level food insecurity rates, but 211 provides more granularity.**

7. The justification for locating this study in Utah need justifying. Presumably it reflects the researchers’ location but a more convincing reason is needed, alongside reflection on the potential transferability of the project findings to other settings.

**This manuscript stemmed from a unique community partnership that the first author had with the Utah’s 211 system (Please see line 88). Although the transferability of the project findings to other settings is difficult to state, we included transcripts from rural and urban areas and also from non-white and diverse ethnicity populations, as described in our updated sampling approach, so that our sample is representative of Utah’s rural/urban structure and racial/ethnic population. Please see line 99. Moreover, our study team concluded that our analysis reached a saturation point of important themes and quotes. Hence, our results are generalizable to other similar settings in regard to rurality and racial/ethnic categories.**

8. Why did the researchers sample 25 calls? What proportion of monthly calls to the 211 helpline is this?

**25 transcripts were chosen as it is a reasonable sample size for qualitative analysis. Please see our response to your concern numbered 1.**

**After analyzing these 25 transcripts, we did not see the emergence of new themes and felt comfortable that we had reached saturation. Please see line 143.**

9. Dates of data collection would be helpful to contextualize the study.

**We had mentioned the time frame in the abstract. We now added this in the method section as well. Please see line 95.**

10. Further information about the sampled calls is needed: what is their average duration (and how does this compare to calls in general)? Did the researchers draw their sample of 25 calls from food-related calls only, or from calls seeking assistance in other areas too? What drove this decision? Did the 25 sampled calls cover only food-related topics, or were other areas of need also discussed? (this feels especially important in light of the analytical theme of the complex social environment and the competing demands shared by callers). Overall in this section I was left feeling very unclear about exactly how the calls were sampled, and why.

**We do not have access to the recordings of calls as a senior community resource specialist at the United Way of Salt Lake transcribed the calls for us and provided us with the transcripts. However, we provided the average and range of word counts in the results section, line 130.**

**These 25 calls are only from food-related calls, as described in the Methods section. While each call can be classified into more than one category based on the discussion during the call (e.g., food, housing, healthcare), our focus in this paper was on calls that had indications of food needs. Meaning that other needs, besides food-related needs, could have been discussed during these 25 selected calls.**

11. Why did the researchers use thematic analysis? This decision needs justifying.

**We used the thematic analysis technique because it is widely used in qualitative analysis and in health-related fields, aimed at interpretation, meaning-making, and using systematic coding procedures. Please see lines 110-112.**

12. What is meant by ‘to roll up the codes into themes’? (p4) Clarity is needed here.

**We revised this sentence and now it reads as “to create a diagram to illustrate and organize codes into themes.”**

13. Given the use of a purposive sample, there is a circularity in reporting descriptive statistics (because the composition of ZIP codes for example was selected by the researcher) that needs acknowledging.

**Please see the updated Table 2 caption and the footnote under Table 2, which reads as “****A purposive sampling procedure was used to select those 25 food-related calls.”**

14. This is not stated explicitly but the information contained in Table 2 implies that some data was missing (eg: age was reported by 8 callers out of 25). A brief account of why some data are missing and reflection on the possible reasons and implications for this is needed.

**At the time of the call, a 211 resource specialist documents a caller’s ZIP Code, gender, primary language, and reason for the call. Other information, such as age, race/ethnicity, and education are not uniformly gathered. Please see lines 132-134.**

15. In Table 2, what is meant by ‘Household member’? Do you mean household size? If so it would be clearer to state this directly.

**We changed that to household size.**

16. Theme 1: Information seeking seems conceptually confused. The quotes listed on page 5 (lines 128-141) actually seem to focus on two themes: information seeking, and callers being referred. These are quite different pathways to seeking help via the 211 helpline and this diversity is not captured in the theme.

**Thanks for raising attention to this matter. We changed the theme’s name to “information seeking and referrals to 211” and we believe that it now better reflects the content of calls within this theme. But please note that for this theme we selected quotes that reflected the reason for their call and how they heard about 211. Please see line 149, where we made this point clear.**

17. a. Some of the quotes listed on p5-6 (focussing on the theme ‘lack of food and informational barriers’) would more appropriately be coded within the second theme of food insecurity. This overlap between themes is concerning and suggests that further analytical work is needed. Some of the quotes included in theme 2 (food insecurity) also overlap with theme 3 (complex social environment). This comment applies particularly to quotes on the theme of competing financial demands. As above, the overlap between themes suggests that further analytical work is needed to identify relevant themes (or at the very least, this fluidity of themes needs acknowledging and reflecting upon).

**We appreciate the point about overlapping themes.** **We chose to organize the themes around a narrative of access, food insecurity as conceptualized by USDA, other social needs, and barriers to resources that contribute to FI. We moved two quotes from the food insecurity theme to the third theme.**

**We have now clarified that Theme 1 included codes related to information seeking and referrals to 211 and that information seeking included the caller’s stated reason for calling (e.g., “I have no food”).**

**In theme 2, we used a strict definition based on monetary needs and according to the USDA’s food insecurity measurement. This theme included quotes related to the lack of money to buy food.**

**In theme 3, complex social needs included quotes relating to other social needs, which contributed to household financial shortages through competing needs. Please note that the quotes within this theme are explaining other social needs that callers are dealing with and there is no mention of lack of money to buy food.**

**And finally in theme 4, callers reported barriers in accessing resources due to ineligibility or lack of transportation. We acknowledge that our themes are interrelated but each theme points to distinct facets of food insecurity represented within the call transcripts; please see line 327-329.**

b. On p6 the authors state ‘In several calls, there is much evidence that callers’ needs have been met’. Who have their needs been met by? Again, for international readers this sort of contextual information is needed.

**Please see the revised sentence in line 196 which reads as “In several calls, there is much evidence that callers’ information needs have been met by the operator.”**

18. The authors state ‘47% of low-income people do not use food pantries due to informational barriers (i.e., lack of information) [25]. Analyzing the transcripts also highlighted the fact that some people are not using government assistance programs like SNAP but they still have unmet food needs’, implying that lack of information might explain why some callers were not receiving SNAP. Yet the analyses highlighted eligibility issues, not lack of information, as a barrier to SNAP use in this sample. This mismatch between the published research and the findings presented here suggest a limited understanding of the study’s findings, implications, and relevance to existing scholarship on the topic.

**While only one caller mentioned the ineligibility for SNAP (as shown in Theme 4), many other callers that had food needs were not using any government assistance programs including SNAP. The 211 specialists sometimes ask a question about using government assistance programs, however, this is not a common practice.**

19. The following sentence (p9) does not make sense: ‘Paying for medical bills, unemployment, disability, increasing costs of gas, grocery, and rent, and the impact of COVID-19 on callers’ mental health are among stressors mentioned by callers that have created an unmet food need a complex problem to address’.

**Please see the revised sentence in lines 311-313.**

20. The authors note that the sample is representative of the Utah population. On what grounds are they making this statement? With my limited understanding of the 211 community helpline I would expect that the sample is one of low-income, vulnerable people, so not representative of the state population at all. If the authors mean that the representative of the urban/rural make up of the Utah population then they ought to state this instead. It would also be helpful to contextualise the findings in relation to the US more generally to help readers appreciate the relevance of this study beyond the immediate study location.

**Please see the revised sentence in line 329.**

**Regarding the transferability of the project finding, please see our response to your concern numbered 7.**

21. The statement ‘Our analyses captured discrete information that was hidden in food-related information seeking calls’ is confusing. In what way was this information hidden? I would encourage the authors to rephrase this assertion.

**Please see the revised sentence in line 334.**

# Reviewer #2

1. More detail is needed about the sampling approach and inclusion criteria that guided selection of the 211 calls that were analyzed. For example, were there quotas for different demographic sub-groups? Was sampling meant to be proportional to some populations (e.g., 211 callers, residents of Salt Lake County)? Were only English-speaking callers eligible? Were the included transcripts from people who called 211 about food assistance, or from people who called about something else (e.g., utility payment assistance) and were then asked about food?

**Thank you for bringing this to our attention. First, we identified food-related calls based on taxonomy (using codes to categorize calls). Among those calls, based on a purposive sampling approach, 25 were sampled based on their location (i.e., metro vs. nonmetro classification of ZIP Codes) to ensure representation of rural residents as they might have different food insecurity experiences. We requested variation in race/ethnicity among callers, similar to Utah’s population. 77% of Utah's population are non-Hispanic white and 14.4% of the population are Hispanic or Latino. Please see lines 94-104.**

**211 provides services in 180 languages using non-English operators and access to a translation line.** **We did not restrict our sample to English-speaking callers, but we do not know whether those callers used a non-English operator or a translation line as we were only provided the transcript of calls. Please see lines 105-107.**

**While each call can be classified into more than one category based on the discussion during the call (e.g., food, housing, healthcare), our focus in this paper was on calls that had some indications of food needs.** **Meaning that other needs, besides food-related needs, could have been discussed during these 25 selected calls. And that is why you see some quotes in the “Complex Social Environment” theme that highlights other needs like school loans or healthcare mentioned by the caller, besides their food needs. Please see lines 96-99.**

2. What is the service area covered by the 211 -- is it all of Utah, or only Salt Lake County? Demographically, those would be quite different.

**The 211 network is available in the U.S. and Canada. Utah’s 211 call center in Salt Lake City covers all of Utah. We added these points in the Methods section.**

3. How long (in word count) were the transcripts? Please report a range and mean. What is meant by "diagram" in Phase 3 of the analysis? What does the diagram visually illustrate? In Phase 4, "vetted" for what?

**We added the mean and range of word counts in the Results section, line 130.**

**Please see the revised Table 1 and the paragraph above it.**

4. Can you provide a sense of the relative frequency of each theme? For example, were some more common than others? Also, how much overlap was there among themes? In other words, presumably one transcript could have elements of multiple themes. Did some themes overlap more than others?

**The theme with the highest number of coded quotes was information seeking and referrals to 211, followed by barriers to resources, complex social environment, and food insecurity. We added this sentence in the Results section, line 140. However, it is important to note that thematic analysis generally does not incorporate counts. Each transcript could contain quotes that belonged to different themes, but this is inherent to any qualitative analysis.**

5. Why did so few callers provide age and education information? What is meant by "household member"? Is this a count of household size? Why did so few callers provide this? Were these demographic variables systematically assessed? Inferred from call content?

**Age, education, and other demographics are not asked frequently. Some of the callers mentioned those voluntarily and we** **inferred them from the contents of the calls, and sometimes the 211 specialists asked those questions, as this is part of their practice (i.e., they select a few callers randomly and ask a more comprehensive set of questions if the caller agrees). However, ZIP Codes are reported for all the calls.**

**“Household member” was changed to “household size”.**
