# Supplementary material for: Indications of food insecurity in the content of telephone calls to a community referral system
Source: PLoS One. 2023 Apr 20;18(4):e0284624. doi: 10.1371/journal.pone.0284624 (PMC10118074; doi:10.1371/journal.pone.0284624)
Supplement: Attachment — Submitted filename: PLoS one re-review march 2023.docx [file pone.0284624.s002.docx]

Indications of food insecurity in the content of telephone calls to a community referral system

PONE-D-22-22201R1

Second review of manuscript, March 2023

The revised paper shows significant improvements in terms of clarifying some key details of the project’s design and methods. However I still stand by my original recommendation that the paper lacks scholarly rigour and as such does not represent a significant or robust contribution to scholarship.

Primarily I am concerned about the way that calls to the 211 service were sampled. In my original review I asked for clarity on a number of points relating to the selection of phone calls for analysis. The author response states that calls were chosen by their non-academic partner and that the research team did not have access to call recordings. The author is therefore stating that they were not responsible for sampling the phone calls which form the primary data for their analyses. I acknowledge that researchers undertaking secondary data analysis will inevitably face constraints associated with this method but the lack of involvement of trained researchers in sampling for this project is a fatal flaw in terms of data quality and academic rigour more generally.

I also have further concerns about the paper’s scholarly rigour:

- In the abstract the authors quantify the sample composition (‘There were 13 calls from metropolitan and 12 calls from nonmetropolitan ZIP Codes. Participants included 5 Hispanic, 8 non-Hispanic, 7 white, and 5 non-whites.), but this is not a meaningful approach when undertaking qual research. Given that purposive sampling was used to determine the sample composition, it is an entirely circular statement that demonstrates a poor understanding of qualitative research approaches
- The authors justify thematic analysis on the basis of its popularity, not in relation to its appropriateness to exploring their research questions. Having been prompted to provide clear justification for this decision, their failure to do so is indicative of a careless approach to research design
- The authors make assertions that are not supported by the data they report. For example, when I queried their statement that ‘In several calls, there is much evidence that callers’ needs have been met’, the revised paper now reads ‘In several calls, there is much evidence that callers’ information needs have been met by the operator’, without providing any data to support this assertion.
- In several places, the authors (apparent) response to reviewer comments is missing from the revised paper. For example, I questioned the meaning of the sentence ‘Our analyses captured discrete information that was hidden in food-related information seeking calls’ and the authors made reference to a revised sentence on line 334. The revised manuscript does not have a line 334 and a text search of the term ‘hidden’ does not reveal any changes to the manuscript. This is either the mark of careless scholarship or a deliberate attempt to mislead the reviewers and editor; either option is very discouraging
- More generally, several author responses to the reviewer comments have not been incorporated into the manuscript, suggesting that the authors have poor knowledge of the purpose of peer review
